# Construction and Characterization of a cDNA Library from Wheat Infected with *Fusarium graminearum* Fg 2

**DOI:** 10.3390/ijms12010613

**Published:** 2011-01-18

**Authors:** Khaled Al-Taweel, W. G. Dilantha Fernando, Anita L. Brûlé-Babel

**Affiliations:** 1 Department of Plant Science, University of Manitoba, Winnipeg, Manitoba R3T 2N2, Canada; E-Mails: khaledta72@hotmail.com (K.A.-T.); anita_brulebabel@umanitoba.ca (A.B.-B.); 2 General Commission of Agricultural Scientific Research (GCASR), Department of Biotechnology, P.O. Box 113 Douma-Damascus, Syria

**Keywords:** 15 acetyl deoxynivalenol (15ADON), 3 acetyl deoxynivalenol (3ADON), cDNA library, *Fusarium graminearum* Fg2, Triticum aestivum

## Abstract

Total RNA from wheat spikes infected with *F. graminearum* Fg2 was extracted and the mRNA was purified. Switching Mechanism at 5′ end of the RNA Transcript (SMART) technique and CDS Ill/3′ primer were used for first-strand cDNA synthesis using reverse transcriptase by RT-PCR. Primer extension polymerase chain reaction was used to construct the double-strand cDNA that was digested by proteinase K, then by *Sfi* I and fractionated. cDNAs longer than 0.5 kb were collected and ligated to λTriplEx2 vector followed λ phage packaging reaction and library amplification. The qualities of both unamplified and amplified cDNA libraries were strictly checked by conventional titer determination. One hundred and sixty five plaques were randomly picked and tested using PCR with universal primers derived from the sequence flanking the vector. A high quality cDNA library from wheat spikes that have been infected by *F. graminearum* was successfully constructed.

## 1. Introduction

Fusarium head blight (FHB), caused by *Fusarium graminearum*, has posed a serious threat to wheat production worldwide [1]. The disease not only lowers grain yield but reduces grain quality as well [1]. Breeding wheat resistant to FHB is one of the best options to minimize crop and grain quality losses [1]. Consequently, the absence of visible host responses or disease symptoms at the early stages following infection has stimulated investigations of the underlying nature of pathogen-host interactions at the molecular level. Understanding the molecular mechanisms underlying these interactions is of primary importance in devising strategies to control diseases. For this purpose, differentially expressed genes (DEGs) analysis is a method of choice. One of the most powerful techniques for such analysis is suppression subtractive hybridization (SSH), and a cDNA library is an essential tool that helps SSH screen the full-length of DEGs [2].

Expression of genes is essential for normal development and pathological processes. Therefore, discovery of DEGs is critical for the understanding of the molecular mechanisms involved in normal and pathological states, as well as providing new insights for discovery of novel genes. Thus, a cDNA library is crucial to pick up the full-length of the genes of interest [3].

Constructing a cDNA library lays basic foundation for finding relevant genes and investigating their functions. Unlike genomic DNA that has introns in it, cDNA contains an open reading frame ready for expression. Therefore, a cDNA library can be used not only to screen the target genes required, but also to express them [4].

A cDNA library can be constructed from *Fusarium*-infected wheat and used to find the relevant genes and interesting proteins associated with the defense mechanism in plant. For example, single strand cDNAs reverse transcribed from mRNA extracted from wheat spikes inoculated with Fg2 can be used to hybridize with excessive cDNAs from healthy wheat plant (control). Then the remainder of the cDNAs (DEGs under the pathogen) can be used as subtracted cDNA probes for screening a cDNA library constructed from *Fusarium*-infected wheat to search for the homologous clones. Using this method, the full-length genes that are exclusively expressed under Fg2, which induces 3 acetyl deoxynivalenol (3ADON), could be obtained.

The sequencing and characterization of cDNA, which represents a direct link to functional genomics, is a powerful means of identifying genetic polymorphisms and is essential for the determination of different gene expressions [5,6]. In addition, the construction of a cDNA library and the improvement of gene cloning methods will help the identification and cloning of the expressed genes under specific conditions. This will further elucidate the molecular mechanism of the plant-pathogen interaction, since the full-length cDNAs of wheat infected with *F. graminearum* Fg2- 3ADON deposited in the public database are limited.

On the other hand, the study of full-length cDNAs remains an indispensable approach for structural and functional genome annotations [7]. Obtaining whole length and an intact cDNA library is often a challenging process. The major challenge every full-length cDNA method tries to resolve is grafting a known sequence at the cap site, so as to be able to prime second-strand polymerization of the cDNA. In some methods, cap-dependent tagging is used as a way of selecting for full-length cDNAs while in others the tag is added on cDNAs previously enriched for molecules extending to the 5′cap [8,9]. A number of enzymatic or chemical tagging have been described, either on single-strand cDNA [10,11], double-strand cDNA [12,13], de-capped mRNA [14–16] or straight on the mRNA cap [17]. In the strategy followed by Sekine and Kato [15] and of Maruyama and Sugano [16], RNAs were dephosphorylated by alkaline phosphatase, decapped by tobacco acid pyrophosphatase and ligated to an oligonucleotide by T4 RNA ligase.

In this study, a cDNA library was constructed using Clontech’s patented SMART (Switching Mechanism At 5′ end of RNA) cDNA technology from wheat inoculated with 3ADON-producing *F. graminearum* Fg2.

## 2. Results and Discussion

### 2.1. Analysis of Total RNA

**Figure 1:**
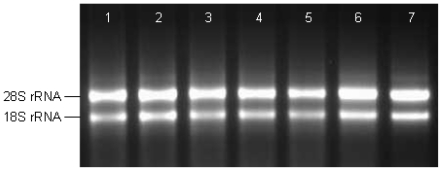
(1.1%) Agarose gel electrophoresis of total RNA. Total RNA extracted from *Fusarium*-infected wheat spikes was fractioned. Lanes 1, 2, 3, 4, 5, 6, and 7 represent samples collected 6, 12, 24, 36, 48, 72hr, and 6 days after-inoculation, respectively. One microliter of RNA was loaded for each sample.

In this study, the ratio of the A_260_/A_280_ for total RNAs were 1.95–2.1, and the average concentration was 750 ng/μL according to the absorbance of ultraviolet light at 260 nm. Total RNAs of post-inoculation spikes were examined by electrophoresis on 1.1% agarose gels (Figure 1) showing two bright bands; 28S rRNA and 18S rRNA where the former was equal to or more abundant than the latter indicating that little or no RNA degradation or contamination occurred during isolation. Therefore, the total RNA isolated from the *Fusarium*-infected spikes was pure, integrated and stable for cDNA library construction.

### 2.2. Analysis of mRNA

**Figure 2:**
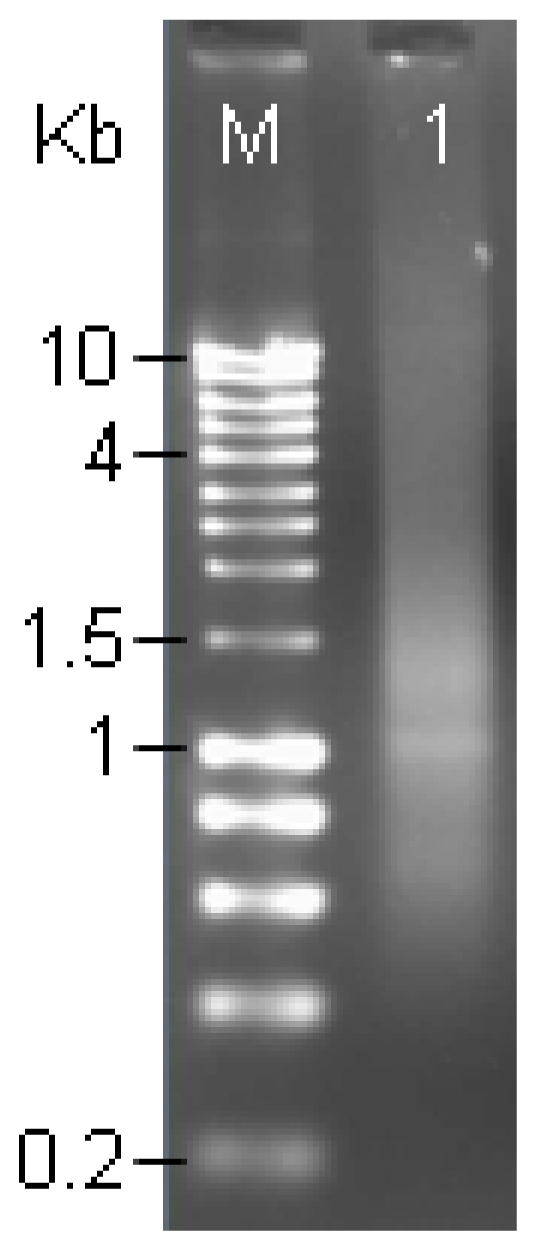
A 1.1% agarose gel electrophoresis of Poly(A)^+^ RNA. A uniform smearing pattern of the Poly(A)^+^ RNA indicates a high mRNA quality purified from total RNA. M: RNA ladder; Line 1: Poly(A)^+^ RNA (0.2 μg).

In this study, the 1.1% agarose gels (Figure 2) showed a distributed smear from 0.4–4 kb. Therefore, a conclusion could be drawn that the total RNA obtained from *Fusarium* infected spikes was of high quality and quantity. The quantity and quality of cDNA is a key to construct high quality cDNA library, but the quality of cDNA is affected by mRNA. In this study, the total RNA was isolated by RNAase kit (Qiagen), and in comparison, this method was found to be more efficacious than other methods to extract RNA [18]. The quantity and integrity of total RNA and mRNA detected by ultraviolet spectrometer and by electrophoresing on an agarose gel was good. Low abundance mRNA (<14 copy/cell) exists in about 30% of all mRNA. According to Clareke-Carbon’s formula, a cDNA library should contain at least 1.7 × 10^5^ independent clones [4], so that a clone derived from low abundance mRNA would be screened out with 99% probability from the library. The capacity of the cDNA library constructed in this study was 5.5 × 10^9^ pfu/mL, which could meet almost all requirements of finding a cDNA clone derived from low abundance mRNA. Furthermore, the recombination efficiency of unamplified and amplified libraries was well over 90%. The unamplified library had no less than 1 × 10^7^ clones which, in principle, was sufficient for including the most mRNA of rarely expressed.

### 2.3. Analysis of Primer Extension Product

**Figure 3:**
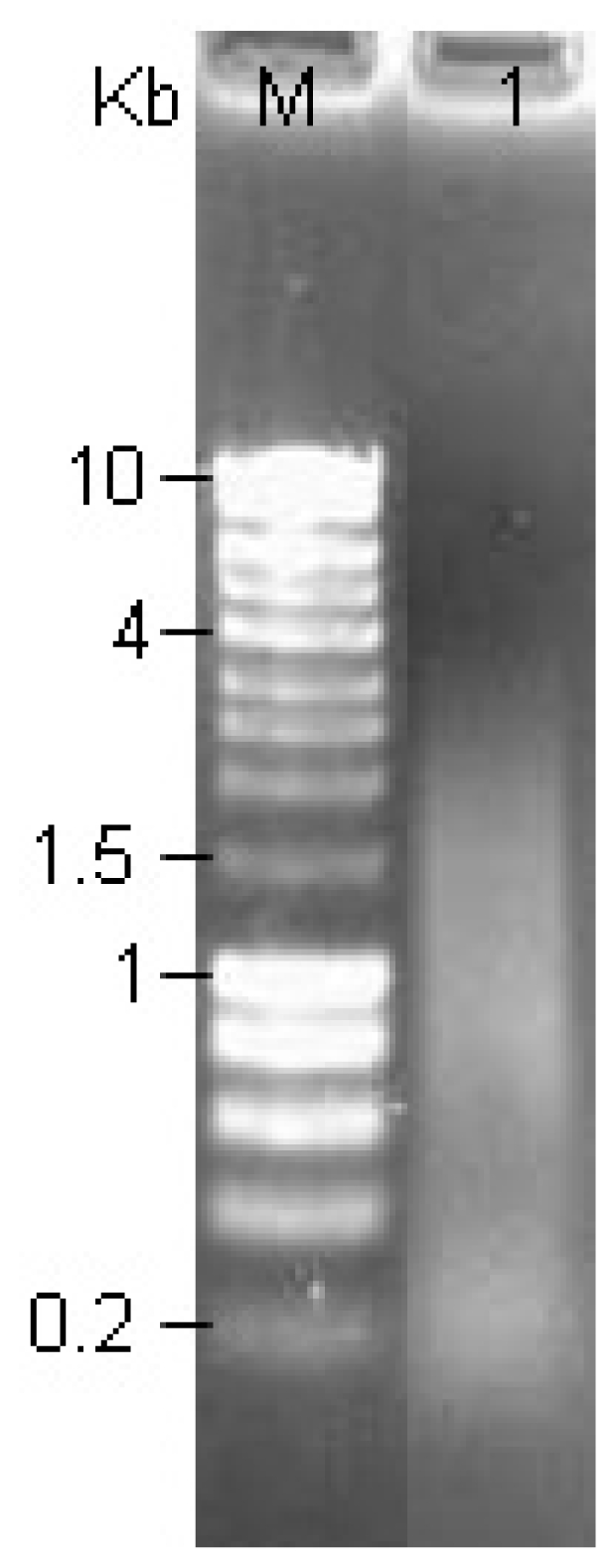
A 1.1% agarose gel electrophoresis of ds cDNA after PCR. One μg (3 μL) of Poly(A)^+^ RNA was used as RNA template with the indicated primers in a first-strand synthesis. 11 μL of ss cDNA served as a template for primer-extension based, second-strand synthesis using 3 thermal cycles. Lane M: HyperLadder marker (5 μL). Lane 1: 5 μL sample of the ds cDNA product showing a smear ranging from 0.2 to 3 kb.

Using 1 μg high quality Poly(A)^+^ RNAs, first-strand cDNAs were synthesized according to the protocol of SMART™ cDNA Library Construction Kit (Clontech, USA). Then, whole volume of the ss cDNA (11 μL) was used to synthesize the ds cDNAs. After 3 cycles, 5 μL of 100 μL was analyzed by agarose gel electrophoresis (Figure 3). The bands of double-stranded cDNAs were dispersed and the length was mainly ranging between 0.2–3 kb.

### 2.4. Analysis of Size-Fractionated cDNA

cDNA-fragments smaller than 500 bp and longer than 3000 bp were eliminated by cDNA fractionation using a CHROMA SPIN-400 column to avoid the library having a preponderance of very small inserts and/or non-recombinant clones.

**Figure 4:**
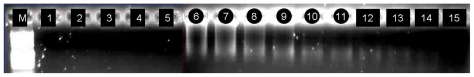
A 1.1% agarose gel electrophoresis of size-fractionated cDNA for removal of small oligonucleotides. Single-drop fractions (15 fractions, 35 μL each) were collected in separated tubes, and 3 μL of each fraction was electrophorised on 1.1% agarose gel at 150 V for 10 min. The lightest lanes (circled numbers 6–11) indicating cDNA peaks were collected for ligationstep. M: HyberLadder marker; Lanes 1–15 are cDNA size-fractionated samples.

To check the profile of the collected fractions, fifteen fractions were electrophorised on 1.1% agarose gel and the desired size fractions were determined (Figure 4), pooled, precipitated and then resuspended in 7 μL water to be ready for ligation step.

The early work of Maniatis [19] provided a foundation for the use of cDNA libraries in molecular and genomic research. Since that time, the construction of cDNA libraries has become one of the most fundamental tools in genomics research. Once constructed, cDNA libraries can be used to identify genes of interest as well as serving as a physical resource for full length clones. Although there are tremendous benefits for the successful generation of a cDNA library, historically the process of cDNA library generation has been inconsistent and laborious. A high percent of 5′-truncated clones may be generated from traditional methods and protocols involving full-length mRNA are labor intensive and susceptible to degradation from RNase. In the current study, with SMART, we could enrich full-length double-stranded cDNAs, generate high-quality cDNA from nanograms of total RNA, and use cDNA for library construction.

### 2.5. Analysis of the cDNA Library

#### 2.5.1. Titering the Unamplified Library and Determining the Percentage of the Recombinants

**Figure 5:**
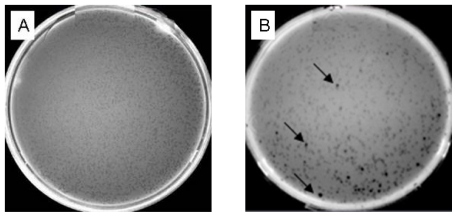

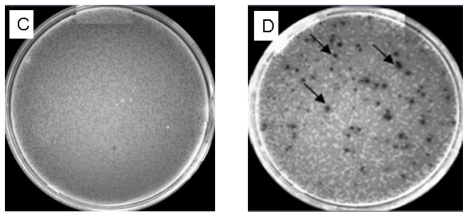
**(A)** The titer of unamplified library. (**B**) Blue and white screening of the unamplified library. Arrows indicate blue plaques. The two plates (**A**, **B**) were inoculated with a 10-fold dilution of packaged phage. Plaques were approximately 1 mm in diameter. (**C**) The titer of amplified library. (**D**) Blue and white screening of the amplified library. Arrows indicate blue plaques. The two plates (**C**, **D**) were inoculated with a 1 × 10^4^ dilution of phage lysate. Bacteriophage: λ lcI857 Sam7. Host: *Escherichia coli* LE392MP.

The titer of the unamplified library corresponding to the concentration ratio of cDNA and λ phage vector (1/10) was 1 × 10^7^ pfu/mL. The recombination clones were analyzed by X-gal, and there were 80 blue plaques and 920 colorless plaques in one plate giving a recombination efficiency of 92% (Figure 5A,B).

#### 2.5.2. Titering the Amplified Library and Determining the Percentage of the Recombinants

The 1:10 diluted packaged lysate was amplified to yield 1 × 10^5^ plaques per 150 mm-plate. Twenty 150 mm-plates were plated counting 2 × 10^6^ independent clones, yet, in most cases, 1 × 10^6^ is representative of the mRNA complexity. The titer of the amplified library was 5.5 × 10^9^ pfu/mL, and the percentage of the recombinants according to the blue and white screening was 94% (Figure 5C,D). These results are in agreement with constructing a library.

### 2.6. Identification of the cDNA Inserts of the Recombinants

**Figure 6:**
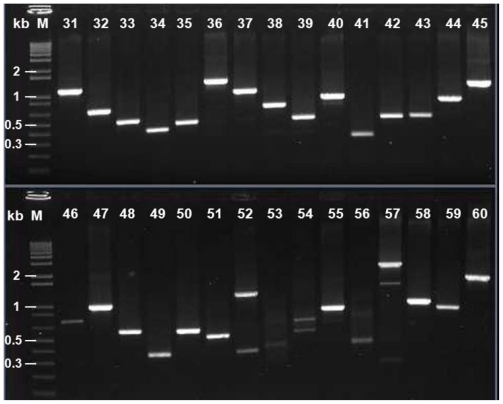
A 1.1% agrose gel electrophoresis of PCR products of cDNA inserts selected randomly (165 plaques) from the amplified cDNA library. Lane M: 1 kb Plus DNA marker (Invitrogen); 31–60: A part of plaques selected randomly and insert-amplified by PCR.

One hundred sixty five plaques were randomly picked and amplified by PCR using the universal primers mentioned earlier. The sizes of PCR products were between 1–2 kb for 110 samples (66.67%), 0.5–1.0 kb for 55 samples (33.33%) and the majority inserts were well over 0.5 kb (Figure 6).

The technology used in this study for full-length cDNA enrichment is robust and only requires less than 1 μg of starting total RNA. By using the MMLV reverse transcriptase, only the 5′-end tagged cDNAs are not prematurely terminated and can be amplified into full-length by an RNA oligo-specific primer [20,21]. The size fractionation process was applied to remove small-molecular weight such as primers, DNA, and dNTPs. The enrichment of the full-length cDNA was achieved by PCR amplification following the cDNA synthesis. Because selection bias could favor the smaller cDNA, fewer PCR cycles were used to minimize such bias as previously suggested [20]. The conventionally constructed cDNA libraries rarely carry cDNA inserts over 2 kb, because the longer transcripts are often easily truncated during cDNA synthesis process, causing size bias against the larger cDNA fragments in cloning process. In this study, cDNA synthesis by primer extension was used as it is ideal protocol for researches who are not limited by their starting material.

FHB disease can cause animal feed refusal/sickness and illness in humans by producing mycotoxins that consist of nivalenol (NIV), deoxynivalenol (DON), 3ADON, and 15 deoxynivalenol (15ADON). Isolates of 3ADON chemotype have been found to produce significantly higher levels of DON than those with a 15ADON chemotype. Therefore, based on the constructed cDNA library, we will be able to have a full length of DEGs and investigate the transcriptome pattern between 3ADON and 15ADON-infected wheat plants using quantitative real-time polymerase chain reaction (This research is ongoing).

## 3. Materials and Methods

### 3.1. Plant Materials

Wheat (*Triticum aestivum* L.) line “RCATTF203/2: Sumai3”, selected from the cross Funo × Taiwan wheat and resistant to FHB, was used in this study. Plants were grown in a controlled environment in a growth cabinet with a 16-h photoperiod and 18/15 °C day/night temperatures. Plant-Prod (20-20-20) all-purpose fertilizer (Brampton, ON, Canada) was applied at a rate of 6 g/L every second week with.

### 3.2. Pathogen and Inoculation

**Figure 7:**
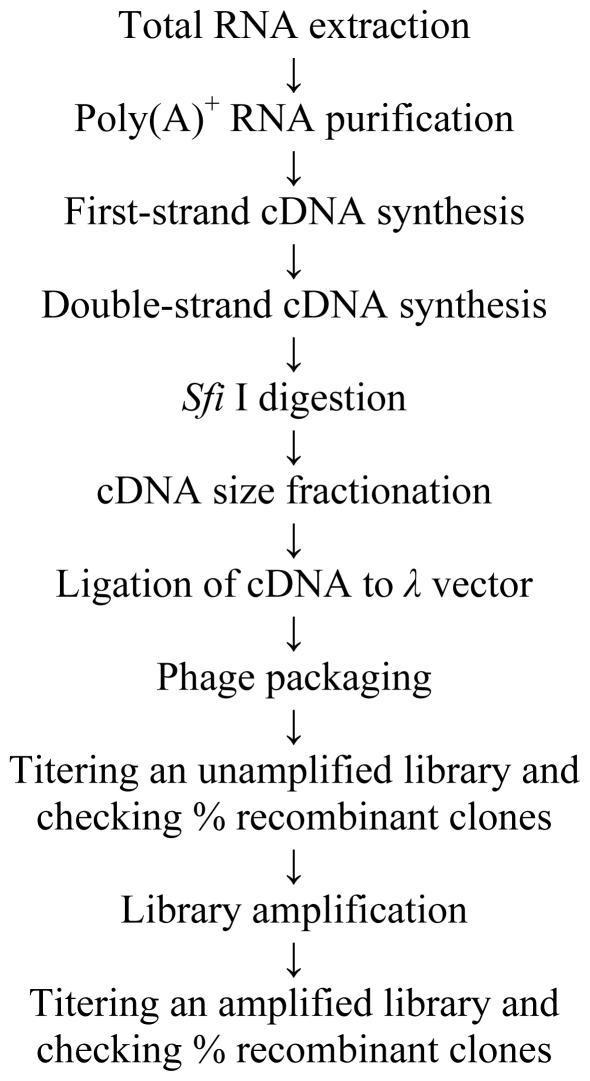
Flow diagram of the cDNA library construction.

A highly virulent isolate of *F.graminearum* Fg2 that produces 3ADON was used for inoculation. Inoculum concentration was 5–10 × 10^4^ conidia/mL. Two florets after the ten basal spikelets were inoculated with 10 μL of a conidia suspension. The inoculated spikes were covered with transparent plastic bags, dried at 60 °C for 72 h in order to standardize humidity content. The infected spikes were harvested 6, 12, 24, 36, 48, 72 h, and 6 days after inoculation, immediately immersed in liquid nitrogen and then stored at −80 °C until processed. Later, the total RNA was isolated from inoculated spikes for the construction of a cDNA library which is summarized in Figure 7.

### 3.3. Extraction of Total RNA

Isolation of high quality total RNA and purification of mRNA are critical steps for constructing a cDNA library. Total RNA was extracted from *F. graminearum* conidia suspension-inoculated spikes. Prior to the extraction of RNA, for each sampling time, three spikes from three different plants were pooled together to reduce the level of biological variation between the samples. Frozen spikes were ground using sterile pestle and mortar with liquid nitrogen into a fine powder. Total RNA was isolated from 100 mg of powder in Eppendorf tube using the silica membrane spin column provided with the RNeasy Plant Mini Kit (Qiagen, Maryland, USA) and subsequently treated with DNase I from RNase-Free DNase Kit (Qiagen, Maryland, USA) to remove genomic DNA, according to the manufacturer’s instructions. Based on the different time of sampling, we obtained seven total RNA samples. The integrity of the total RNA was analyzed using 1.1% agarose gel electrophoresis. The concentration and purity of the total RNA were determined by spectrophotometry (Ultrospec 3100 pro, biochrom, Cambridge, England) at 260 and 280 nm wavelengths.

### 3.4. Purification of mRNA

The total RNAs obtained from different post-inoculation times (7 periods) were combined in one tube to get a pooled total RNA of *F. graminearum* infected spikes representing all the expressed genes under *Fusarium*-infection up to 6 days. Poly(A)^+^ RNA was purified from the total RNA by utilizing Promega’s MagneSphere technology using the PolyATract mRNA Isolation System (Promega, Madison, WI) according to the manufacturer’s instructions. Quality of mRNA was estimated using 1.1% agarose gel electrophoresis. Intact plant Poly(A)^+^ RNA usually appears as a smear between 0.5–3 kb with faint 28S and 18S rRNA bands. The pooled Poly(A)^+^ RNA was used as template for constructing a cDNA library using SMART™ cDNA Library Construction Kit (Clontech, USA) according to the manufacturer’s specifications with the following modifications:

### 3.5. cDNA Synthesis

#### 3.5.1. First-Strand

cDNA synthesis: First-Strand cDNA was synthesized according to the protocol of SMART™ cDNA Library Construction Kit (Clontech, USA).

#### 3.5.2. Amplification of cDNA by Primer Extension

Eleven μL First-Strand cDNA, deionized H_2_O, Advantage 2 PCR Buffer, dNTP Mix, CDS 1II/3′ PCR primer (5′-ATTCTAGAGGCCGAGGCGGCCGACATG-d(T)_30_N_−1_N-3′, 5′ PCR Primer (5′-AAGCAGTGGTATCAACGCAGAGT-3′), and Advantage 2 Polymerase Mix were added into a new pre-chilled 0.5 mL Eppendorf tube and was amplified by the following program: 72 °C/10 min; 3 cycles of 95 °C/20 s, 68 °C/8 min. Five μL of the PCR product was taken for analysis using 1.1% agarose/EtBr gel.

#### 3.5.3. Proteinase K Digestion

To inactivate the DNA polymerase activity, 50 μL of amplified ds cDNA and 2 μL of proteinase K (20 μg/μL) were added into a 0.5 mL sterile Eppendorf tube and incubated at 45 °C for 20 min.

#### 3.5.4. PCR Product Purification

PCR product purification was carried out according to the protocol provided. Finally, 79 μL of deionized H_2_O was added to resuspend the pellet.

#### 3.5.5. *Sfi* I Digestion

To generate cohesive ends to link to λ phage for constructing a directional cDNA library, *Sfi* I digestion was applied according to the manufacturer’s instructions.

#### 3.5.6. cDNA Size Fractionation by CHROMA SPIN-400

To efficiently remove low-molecular weight cDNA fragments, small DNA contaminants, and unincorporated nucleotides from the cDNA, the cDNA size fractionation was carried out using CHROMA SP1N-400 columns according to the protocol of CHROMA SPIN-400. Fifteen fractions were collected in the separated tubes and 3 μL of each were fractionated by 1.1% agarose gel. The peak fractions were determined by visualizing the intensity of the bands under UV; the first six tubes of fractions containing cDNA were put together into a clean 1.5 mL eppendorf tube. Sodium acetate, glycogen and 95% ethanol (−20 °C) were added and placed in −20 °C freezer overnight and then centrifuged. The supernatant was carefully removed, and the pellet was washed with 70% ethanol and vacuum-dried. Seven microliter deionized H_2_O was added to resolve the pellet, which was then kept at −20 °C.

### 3.6. Construction of a cDNA Library

#### 3.6.1. Ligation of cDNA to λTriplEx2 Vector

cDNA and λTriplEx2 vector (Clontech, USA) were ligated based on the concentration ratio (1/10: cDNA/vector); the reaction mixture of cDNA, λTriplEx2 vector, ligation buffer, T4 DNA ligase, ATP and deionized H_2_O was incubated at 16 °C overnight.

#### 3.6.2. λ Phage Packaging

λ phage packaging *in vitro* for the ligation reaction to produce unamplified cDNA libraries was set up according to the protocol of MaxPlax™ Packaging Extract (Epicentre, USA), and the product was stored at 4 °C.

#### 3.6.3. Titering the Unamplified Library

XL1-Blue working stock plate was prepared using LB/tetracycline, from which a single isolated colony was picked up and used to inoculate LB/MgSO4/maltose broth to prepare the XL1-Blue overnight culture. The culture was centrifuged and the pellet was resuspended in MgSO_4_. A 1:10 dilution of the packaging product was made, from which 1 μL of the diluted phage was taken and added to 200 μL of the XL1-Blue overnight culture. The phage was allowed to be pre-adsorbed at 37 °C for 20 min. Afterward, the titering protocol was applied according to the manufacture’s instructions. The plaques were counted and the titer of the phage (pfu/mL) was calculated as:

$$\text{pfu}/\text{mL}=(\text{number }\text{of plaques})\times (\text{dilution factor})\times (10^{3}\;\mu \text{L}/\text{mL})/(\mu \text{L of diluted phage plating}).$$

#### 3.6.4. Determining the Percentage of Recombinant Clones in Unamplified Library

Three mL of melted LB/MgSO_4_ top agar (45 °C) was added to mixture of 200 μL of the XL1-Blue overnight culture, 1 μL of the diluted phage, 50 IPTG stock solutions (0.1 mol/L) and 50 μL X-gal stock solutions (0.1 mol/L) into a sterilized tube and then poured onto 90 mm LB/MgSO4 agar plate pre-warmed to 37 °C. After cooling the plates were inverted and incubated at 37 °C overnight. The ratio of white plaques (recombinants) to blue plaques (non-recombinants) rapidly estimated the recombination efficiency. The percentage of the recombinants was calculated as:

(Number of white plaques)×(100%)/(The total plaques“white+blue”).

#### 3.6.5. Library Amplification

The 1:10 diluted packaged lysate that yields 1 × 10^5^ plaques per 150 mm plate was put into a 15 mL sterilized tube with 500 μL of XL1-Blue overnight culture and incubated in a 37 °C water bath for 20 min. After 10 mL of melted LB/MgSO_4_ top soft agar was added into each tube (20 tubes of 15 mL volume), the mixture was poured onto 150 mm LB/MgSO_4_ agar plates pre-warmed to 37 °C. The 20-plates were inverted after being cooled and incubated at 37 °C overnight. Eight mL of lx lambda dilution buffer was added to each plate. The plates were stored at 4 °C overnight to allow the phage diffuse into the buffer, and then incubated on a platform shaker at 50 rpm at room temperature for 6 h. The phage lysates were pooled into a sterile beaker, and extra 2 mL of buffer was added into each plate to dislodge remaining phage from the agar. Five percent final concentration of chloroform was added into the pooled lysate and then poured into a sterile 50 mL polypropylene screw-cap microcentrifuge tube, vortexed for 2 min, and centrifuged at 7000 rpm for 10 min. The supernatant was transferred into another sterilized 50 mL centrifuge tube, and Dimethyl sulfoxide (DMSO) was added (final concentration 7%). The amplified library was put into 1.5 mL sterilized microcentrifuge tubes and stored at −80 °C.

### 3.7. Identification of the Amplified Library

#### 3.7.1. Titering the Amplified Library

Ten μL of 1 × 10^4^ diluted phage and 200 μL of the XL 1-Blue overnight culture was added into a 15 mL sterilized tube, and the phage was allowed to be adsorbed at 37 °C for 20 min. Four mL of melted LB/MgSO_4_ top soft agar was added into the tube, and the mixture was poured onto a 90 mm LB/MgSO_4_ agar plate preheated to 37 °C. The plate was cooled, inverted and incubated at 37 °C overnight. The plaques were counted and the titer of the phage (pfu/mL) was calculated as described above.

#### 3.7.2. Determining the Percentage of Recombinant Clones of the Amplified Library

The percentage of recombinant clones in amplified library was determined in the same way as that for the unamplified library as mentioned earlier.

#### 3.7.3. Identification of the cDNA Inserts of the Recombinants

One hundred and sixty five plaques were randomly picked using sterilized toothpicks from plate into PCR tube with 15 μL deionized H_2_O and incubated at 100 °C for 10 min. Invitrogen DNA polymerase, buffer, dNTP mixture, upstream primer (provided with the kit)—sequence 5′-CTCCGAGATCTGGACGAGC-3′ and downstream prime; sequence 5′-TAATACGACTCACTATAGGG-3′—were added into the tube. PCR was then carried out according to the following program: 95 °C for 3 min; 94 °C/30 s, 55 °C/30 s, 72 °C/2 min for 30 cycles; 72 °C for 7 min. PCR products were checked by running 1.1% agarose gel alongside DNA marker.

## 4. Conclusions

A full-length cDNA library was constructed using *Fusarium*–infected wheat with SMART Primer Extension-PCR techniques. A scheduled cloning of the full-length genes of *Fusarium*-cDNA library, differentially expressed genes resulted from SSH and functional analyses are underway. The full-length cDNA library constructed from the infected wheat spikes conformed to the requirements of a standard library. This library provided a useful resource for the functional genomic research of the *Fusarium*-infected wheat.
